# Novel Fabrication Technology for Clamped Micron-Thick Titanium Diaphragms Used for the Packaging of an Implantable MEMS Acoustic Transducer

**DOI:** 10.3390/mi13010074

**Published:** 2021-12-31

**Authors:** Lukas Prochazka, Alexander Huber, Michael Schneider, Naureen Ghafoor, Jens Birch, Flurin Pfiffner

**Affiliations:** 1 Department of Otorhinolaryngology, Head & Neck Surgery, University Hospital Zurich, University of Zurich, 8091 Zurich, Switzerland; alex.huber@usz.ch (A.H.); flurin.pfiffner@usz.ch (F.P.); 2 SwissNeutronics AG, 5313 Klingnau, Switzerland; michael.schneider@swissneutronics.ch; 3 Department of Physics, Chemistry and Biology (IFM), Linköping University, 581 83 Linköping, Sweden; naureen.ghafoor@liu.se (N.G.); jens.birch@liu.se (J.B.)

**Keywords:** implantable acoustic transducer, packaging, titanium/platinum multi-layer diaphragm, polymer sacrificial material, DC magnetron sputtering, intrinsic stress, stress measurement, stress relief

## Abstract

Micro-Electro-Mechanical Systems (MEMS) acoustic transducers are highly sophisticated devices with high sensing performance, small size, and low power consumption. To be applied in an implantable medical device, they require a customized packaging solution with a protecting shell, usually made from titanium (Ti), to fulfill biocompatibility and hermeticity requirements. To allow acoustic sound to be transferred between the surroundings and the hermetically sealed MEMS transducer, a compliant diaphragm element needs to be integrated into the protecting enclosure. In this paper, we present a novel fabrication technology for clamped micron-thick Ti diaphragms that can be applied on arbitrary 3D substrate geometry and hence directly integrated into the packaging structure. Stiffness measurements on various diaphragm samples illustrate that the technology enables a significant reduction of residual stress in the diaphragm developed during its deposition on a polymer sacrificial material.

## 1. Introduction

The fast growth of the telecommunication market over the last two decades has enabled high investments in the research and development of MEMS transducers such as gyros, accelerometers, and acoustic transducers. The result are off-the-shelf MEMS transducers with a miniature size, low cost, and low power consumption, but at the same time with a sensing performance that meets the requirements of most electronic devices for daily use [1]. In the medical field, implantable sensor solutions play an increasingly important role, but for most applications, the market is too small to justify the required high investment in the development of application-specific, high-performance MEMS transducers that are designed to meet the stringent requirements for the biocompatibility and reliability of implantable devices [2,3]. If so, an eligible solution could be to use off-the-shelf MEMS transducer chips and the application-specific integrated circuit (ASIC) and package them in a customized, biocompatible, and hermetic enclosure that protects the delicate MEMS parts and electronics against the harsh environment in a living subject and vice versa. However, such an approach involves several design difficulties if applied to an acoustic MEMS transducer.

**Figure 1:**
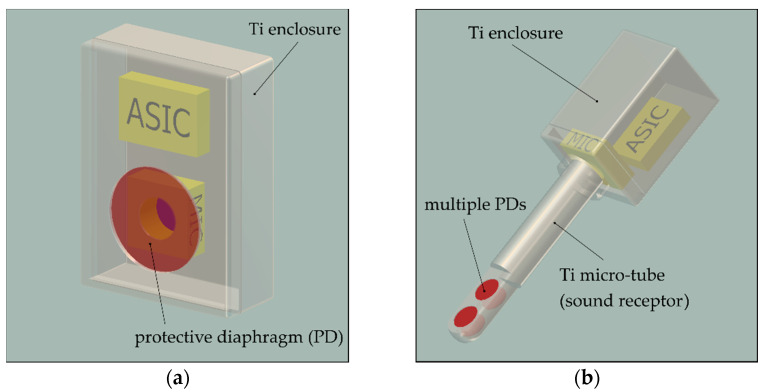
Schematic drawings of a MEMS microphone and the corresponding ASIC packaged in a biocompatible and hermetic Ti enclosure with a PD that can transfer the sound energy to the MEMS transducer. (**a**) A generic enclosure geometry with one larger PD located directly in front of the MEMS microphone diaphragm; (**b**) a more specific enclosure geometry that was developed for an intracochlear acoustic receiver [6]. Four PDs are integrated into a tube-like part of the enclosure to allow the recording of the liquid-borne sound pressure in the human inner ear with sufficiently high receiving sensitivity.

Most acoustic MEMS transducers use a thin flexing element (usually a clamped diaphragm) to capture the smallest pressure fluctuations in the surrounding fluid (receiver) or to radiate sound into the surroundings (transmitter) [4,5]. As soon as the transducer is installed in a hermetic enclosure, the direct access to the surroundings is interrupted. To overcome this problem, a flexible diaphragm-like element must be integrated into the protecting shell that can transmit acoustic sound energy into the transducer’s interior or the surroundings (cf. Figure 1a). We call this design element the protective diaphragm (PD). The PD adds to the mechanical impedance of the MEMS transducer and hence reduces the receiving (or transmitting) sensitivity. Therefore, it must be designed to exhibit maximum compliance and must be arranged close to the transducer’s diaphragm to reduce the volume that couples the two flexible elements. Another difficulty of a hermetic encapsulation of the acoustic transducer is that static pressure equalization between the surroundings and the interior of the packaging structure cannot occur. Excessive loading of the PD by ambient pressure changes can lead to a varying or not tolerable loss in sensing performance. A deeper discussion of that problem and a proposal for a system that can maintain pressure equalization in a hermetically sealed microphone system are given in [6].

To maximize the PD’s mechanical compliance, several design parameters characterizing the PD geometry (size and thickness) and material (Young’s modulus) can be adjusted under the consideration of different design constraints. The PD area is often restricted by the miniature size of the transducer. The requirement for a long-term hermetic encapsulation of a typical implantable transducer restricts the material choice to metals and ceramics and makes it difficult to reduce the PD thickness below 1 µm [7]. Fabrication-related properties of the PD, such as residual stress or the number of pinholes or voids within the PD structure, have either a direct impact on the PD stiffness or augment, for instance, the lower PD thickness limit.

A practical example of a packaging concept that illustrates these design guidelines is the intracochlear acoustic receiver (ICAR), which is currently under development by our group [6,8]. The receiver is designed to be used as an implantable microphone solution for a fully implantable cochlea implant. It is built upon a commercially available MEMS condenser microphone with a customized Ti enclosure that exhibits four circular PDs with a diameter of 0.6 mm and 1 µm thickness. Multiple PDs free of intrinsic stress are required to minimize the loss in sensing performance caused by the encapsulation of the MEMS transducer. Using a theoretical model, a loss in sensitivity of approximately 10 dB was predicted below the resonance operation of the sensor at 3.5 kHz [6]. The inertia of the PDs that are in contact with the perilymph (similar to water) in the inner ear defines the resonance and limits the bandwidth of the ICAR to approximately 6 kHz. To enable sound pressure recording in the tiny human inner ear (cochlea), the PDs are arranged in a pair configuration and on opposite sides within the tip region of a tube-like part of the enclosure (cf. Figure 1b). Titanium is chosen as the enclosure material due to its excellent biocompatibility, hermeticity properties, and inherently higher fracture toughness than ceramics or typical semiconductor materials [9,10]. In addition, it allows the combination of micro- and macro-machining processes such as thin film deposition and etching combined with milling, turning, and welding processes.

The fabrication of micron-thick and sub-millimeter large Ti diaphragms suspended in a Ti structure with a complex geometry is a challenging task. Cold-rolled 1 µm thick Ti foils are commercially available (American Elements, Los Angeles, CA, USA), but the fixation of such a thin foil on the carrier by micro-laser welding or mechanical clamping or bonding is not feasible due to size, mechanical stability, and hermeticity constraints. Mechanical structures with such small dimensions are usually fabricated based on MEMS fabrication technology, which is mostly applied on semiconductor materials such as single-crystal silicon (SCS). MEMS condenser microphones, for instance, which exhibit a sub-micron thick sensing diaphragm sitting on an SCS support and separated from the rigid backplate by few microns, are fabricated based on bulk and surface micromachining technology [11,12].

Various research papers report bulk and surface micromachining processes for Ti as substrate material [13,14,15,16,17]. The most common bulk micromachining process for Ti that is currently widely commercially available is a wet etching technology, called photochemical machining (PCM) [15]. The process is isotropic and thus limits the minimum feature size to approximately the metal sheet thickness if the sheet needs to be etched through [18]. Another bulk micromachining technology for Ti was developed by Aimi et al. [13]. It is a highly anisotropic dry etching technique called the metal anisotropic reactive ion etching with oxidation (MARIO) process. The process allows users to etch straight sidewalls with a high-aspect-ratio and features on the micrometer scale using common dry etching equipment. Bulk micromachining should be better suited than surface micromachining to fabricate a highly compliant PD structure from a Ti substrate because surface micromachined mechanical structures often exhibit high residual stress and hence show high stiffness, high mechanical failure, and high batch to batch variation [19]. However, to obtain a 1 µm thick PD by bulk micromachining, the etch process needs to be stopped very precisely as soon as the targeted etch depth is reached. A time etch stop is not accurate enough to obtain uniform and repeatable structures with micron thickness. Etch stop techniques, as known from the wet etching of SCS (e.g., doping or electrochemical control [20]), which might provide the required etch stop accuracy, do not exist for the PCM of Ti to the best of our knowledge.

To fabricate highly accurate Ti structures at micrometer-scale, bulk and surface machining need to be combined, similar to the case of MEMS fabrication based on semiconductor materials. Such an approach applied to PD fabrication would start with an electrochemically polished Ti substrate with a reactively sputtered TiO_2_ layer covered also with a sputtered structural Ti layer that forms the PD element. The PDs are released from the substrate’s rear side using the MARIO process with the oxide layer as a precise etch stop. The remaining oxide layer is stripped to end up with a pure Ti PD. The process looks simple at first glance, but technologies such as the MARIO process and also the electrochemical polishing of Ti are typically not well established in commercial or research fabrication labs (clean rooms). Creating a more complex enclosure geometry with multiple PDs, as required for the ICAR, creates another difficulty associated with this fabrication approach that is limited to 2D substrates. Thermal compression bonding might be a solution to combine multiple already processed Ti layers, but the compatibility of that quite rough bonding process with the delicate PDs is questionable. To overcome the listed difficulties would require a high process development effort in well-equipped clean-room fabrication facilities, which is associated with high development costs. Therefore, we have developed an alternative fabrication approach for thin diaphragms on a Ti supporting structure that comprises commercially available fabrication processes and processes that can be performed outside of clean-room facilities. In contrast to dry etching techniques, the new fabrication technology can be applied on unpolished substrates with a complex 3D geometry and, therefore, provides more freedom in the design of packaging structures for acoustic MEMS transducers.

The approach is based on the deposition of the structural Ti layer forming the PD on a low-temperature decomposable polymer sacrificial material (SM) as a temporary support. So far, we could confirm the feasibility of producing robust 1 µm thick Ti diaphragms on unpolished substrates with 2D and 3D geometry using this new fabrication approach [6]. We could also show that the method incorporates a stress relief mechanism that originates from a change in the PD’s surface geometry upon releasing from the SM.

The present paper introduces the novel diaphragm fabrication method applied on 2D Ti substrates that belong to a testbed developed for process development and the investigation and testing of the fabricated PDs. The other focus is related to the stress-relief mechanism, which is demonstrated and analyzed on numerous PDs. The paper concludes with a discussion of the results and ongoing studies that are being conducted to obtain robust and hermetic PDs with vanishing residual stress.

## 2. Materials and Methods

### 2.1. Diaphragm Fabrication

#### 2.1.1. Process Overview

**Figure 2:**
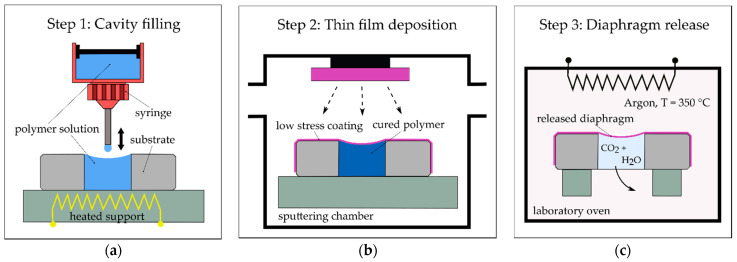
General overview of the diaphragm fabrication process. (**a**) Step 1, filling process: sacrificial material (SM) is added to the substrate; (**b**) Step 2, thin film deposition: metallic coating that represents the diaphragm is deposited on the substrate using chemical vapor deposition; (**c**) Step 3, diaphragm release: the diaphragm is released from the SM during a thermal treatment process.

The process is applied on a substrate with pre-fabricated cavities or through-holes that define the outer size and geometry of the diaphragms. In the first step, the cavities are filled with a fully decomposable polymer sacrificial material (SM) such as polypropylene carbonate (QPAC, Empower Materials, New Castle, DE, USA) dissolved in a solvent (cf. Figure 2a). The solution is added to the cavities using standard adhesive dispensing equipment. For filling, the substrate is mounted on a heated carrier that properly seals the bottom side of the through-holes. Because most of the volume of the added SM (dissolved state) is lost during the curing process, the filling is conducted in multiple steps. The elevated processing temperature of 85 °C enhances the curing process and hence reduces the processing time. After the cavities are filled with SM, a thin metallic film, which forms the diaphragm, is deposited on the front surface of the substrate using physical vapor deposition (PVD) technology such as magnetron sputtering (cf. Figure 2b). Finally, the polymer SM is fully decomposed during thermal treatment in a vacuum or an inert gas atmosphere, releasing the diaphragms from the SM (cf. Figure 2c). The used SM (QPAC) requires a temperature of 350 °C for a full decomposition in various atmospheres, even in a vacuum, forming carbon dioxide and water.

#### 2.1.2. Substrate

**Figure 3:**
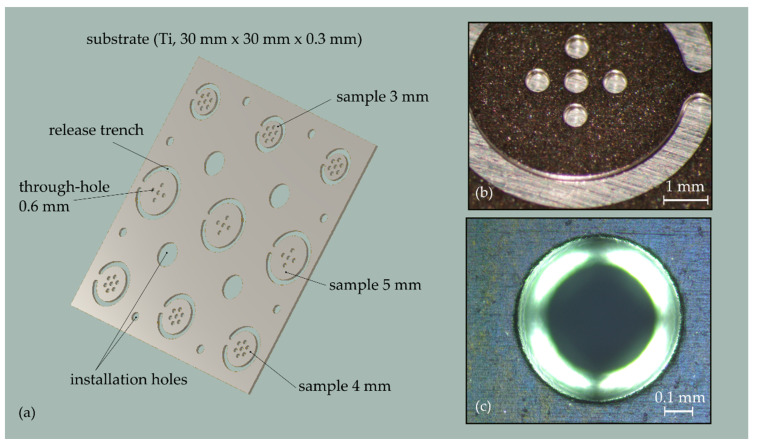
(**a**) CAD drawing of the substrate specifically designed for the development of the diaphragm fabrication process; (**b**) optical microscope image of one of the nine samples on a substrate containing five through-holes; (**c**) microscope image showing the etch profile of a 0.6 mm large through-hole.

The development of the process for diaphragm fabrication was carried out by experimental means, requiring a larger amount of test samples. Accordingly, the substrate was designed to keep the complexity of substrate and diaphragm fabrication small and to enable the simple testing and inspection of the diaphragms. The substrate is a 30 mm × 30 mm large Ti plate with a thickness of 0.3 mm (cf. Figure 3a). It carries 57 through-holes with a circular geometry and a diameter of 0.6 mm. The through-holes are arranged in equally distributed groups of seven and five holes on the substrate. Each group is surrounded by a release trench which forms circular samples with diameters of 3, 4, and 5 mm. Only a narrow connecting bar holds the sample in place during processing on the substrate level and prevents excessive mechanical stress being caused by the handling of the thin substrate, which might potentially damage the delicate diaphragms. Different sample diameters were considered to account for sample integration on different testing and inspection equipment. The diaphragm size of 0.6 mm was chosen to match the corresponding dimensions of the implantable microphone (ICAR). The substrate was fabricated by photo etching (TiME process, Advanced Chemical Etching, Ltd., Telford, UK) of a 0.3 mm thick unalloyed commercially pure Ti sheet from grade 2. The substrate thickness of 0.3 mm results from the trade-off between the minimum feature size that can be etched for a specific substrate thickness and the need for a sufficiently high mechanical stiffness of the substrate. Figure 3b,c are optical microscope images showing the etch profiles of the through-holes with a uniform, slightly rounded edge geometry and sided walls with only a small etch cusp at half the profile depth.

#### 2.1.3. Filling Process

The filling process was performed in a dust-free environment under a laminar airflow hood. QPAC 40 (Empower Materials, New Castle, DE, USA), a low-temperature decomposable polymer, was chosen as the SM for diaphragm fabrication. The granulate (20 wt.%) was dissolved in γ-Butyrolactone (TCI GmbH, Eschborn, Germany). The solution was filtered using a Whatman GD/X syringe filter (GE Healthcare, Chicago, IL, USA) with a regenerated cellulose membrane and a pore size of 0.45 µm. A pneumatic dispenser (PDS 7350, Poly Dispensing Systems, Orgeval, France) and a tiny dispensing needle (size 32 G) were used to control the amount of added QPAC solution. During the dispensing of the SM, the tip of the needle was partially inserted into the cavity or positioned slightly above the cavity opening. The needle position was set by visual observation through a surgical microscope (Wild M650, Leica AG, Heerbrugg, Switzerland) at the highest magnification (40×). The up and down movement of the syringe was adjusted using a manually controlled motorized micro-positioning unit (M-443, Newport, Irvine, CA, USA + T-NA08A50, Zaber Technologies Inc., Vancouver, BC, Canada). Going from one cavity to the other, the substrate mounted on the carrier was manually moved in the lateral direction on a stable support plate.

**Figure 4:**
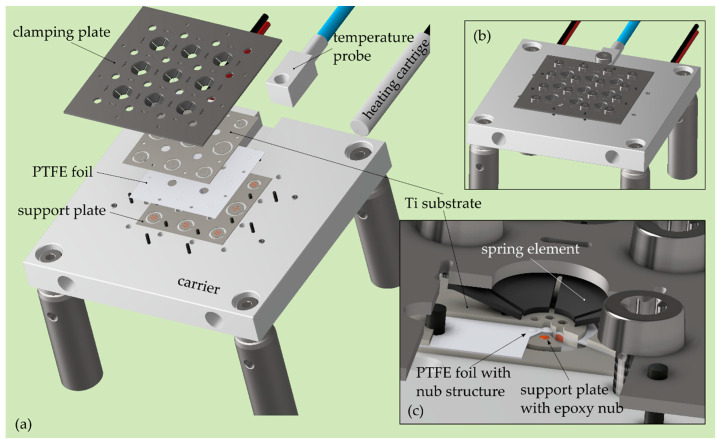
CAD drawings of the instrumented substrate carrier used for the filling process. (**a**) Exploded view showing the different plates for sealing and clamping of the substrate and the heating system of the carrier; (**b**) carrier with the substrate and heating system installed; (**c**) zoomed view on one sample of the substrate illustrates the sealing and clamping mechanism.

Small doses of SM were successively added to each cavity to prevent the solution from flowing over the cavity boundary. The filling process was conducted at an elevated temperature of 80 °C to enhance the evaporation of the solvent, causing a high volume loss of the added material. The filling was therefore repeated several times before the actual curing was performed. During curing at 130 °C for 1 h in a vacuum (>−0.8 bar) atmosphere, the remaining solvent and entrained air were removed from the polymer. At 130 °C, QPAC40 becomes a melted mass of low viscosity, forming by capillary forces a free surface at the cavity rim, which is smooth, uniform, and has a concave shape. The surface curvature of the polymer filling is a critical parameter for stress relief in the diaphragm after it is released from the SM (cf. Section 3). During a repetitive filling and curing process (up to 10 cycles), an optimum surface curvature is adjusted by adding the right amount of SM to the cavity. The substrate was heated using two 100 Watt cartridge heaters installed in the aluminum substrate carrier at a maximum heating rate of approximately 10 °C/min (cf. Figure 4). The cooling rate was mainly controlled by the laminar airflow. A digital PID temperature controller (KS20, West Control Solutions, Kassel, Germany) and a PT1000 temperature probe were used to control a predefined substrate temperature.

Proper sealing of the bottom side of the through-holes is crucial to maintain high filling repeatability and short processing times. The sealing was achieved with a circular PTFE nub (height: 0.1 mm, diameter bottom area: 0.6 mm) pressed against the bottom rim of the through-hole (cf. Figure 4). PTFE was chosen to prevent adhesion between SM and the nub. In addition, PTFE shows high chemical and temperature resistance and, due to the low hardness, it can adapt to any irregularities of the rim geometry. The nub structure was created by adding a 0.1 mm thick PTFE foil as a separator between the substrate and a supporting plate. The latter plate has the same geometry as the actual substrate, but the through-holes are filled with high-temperature epoxy adhesive. The amount of the epoxy was precisely adjusted using the aforementioned dispensing system to create a surface topography on the supporting plate, which was used to stamp the corresponding nubs into the PTFE foil. A laser-cut stainless-steel plate (thickness 0.5 mm) uniformly clamped the whole stack of the individual plates down to the carrier using 16 M2.5 screws. The clamping force was induced at the border of each circular sample plate of the substrate using spring elements (lock washer) attached to the clamping plate. The support plate was reusable, whereas the PTFE foil was replaced when a new substrate was installed on the carrier.

#### 2.1.4. Thin Film Deposition

The diaphragms considered in the present study were made from a 1 µm thick Ti monolayer and titanium/platinum multilayer coatings (Ti/Pt ML). They were deposited on the pre-processed substrate by physical vapor deposition (PVD) in an industrial inline DC magnetron sputter facility Z600 (Leybold Heraeus, Steinhausen, Switzerland) at the company SwissNeutronics AG in Switzerland (Klingnau, Switzerland). To provide good thermalization of the substrate during the deposition, the substrate was screwed down on the carrier plate of the sputtering device. High-purity Ti (PK500 6 mm) and Pt (PK75 2 mm) targets were used for the deposition process. A distance of 61 mm between the substrate and cathode was maintained for all deposition runs. The argon pressure or flow rate were kept constant at 1.2 × 10^−3^ mbar or 55 sccm, respectively. These sputter gas settings were used in literature to produce 1 µm thick Ti layers with low tensile stress [21]. A deposition rate for Ti of approximately 1.2 nm/s and 1.5 nm/s for Pt were achieved with a sputtering power of 1000 W (Ti) and 200 W (Pt), respectively. To prevent excessive load acting on the growing film as a result of the thermal expansion of the SM, it was crucial to keep the substrate temperature during the initial phase of the deposition process well below 40 °C, which is the glass transition temperature of QPAC 40. Using a system for time-resolved substrate temperature monitoring on the Z600 sputtering facility enabled the evaluation of the required low-temperature deposition process. Reverse sputtering, which is described by Tsuchiya et al. 2005 [21] as a mechanism to reduce residual stress in thick (>0.5 µm) Ti films, is not compatible with a polymer SM due to the excessive heating of the substrate during this process step. The active cooling of the substrate might be a solution to mitigate that problem. However, Ti/Pt ML deposition is regarded as an alternative approach for lowering residual stress that is compatible with a low-temperature deposition process and fulfills biocompatibility requirements. In an ideal multilayer system, the two materials exhibit residual stress with opposite signs, a stress level, and layer composition that in combination produce a stress-free multi-layer coating [22]. The fact that zero stress prevails only for a specific design temperature is less critical for an implant that operates in the human body at a constant temperature. We want to emphasize that the investigations of residual stress formation in multilayer coatings are still ongoing and not the focus of the present paper.

For a comparison of the residual stress estimated from the measured diaphragm stiffness data, stress measurements based on the deflection method applied on an SCS test beam were conducted as well. The coated silicon test beam with a certain curvature was placed on a precision edge, and the resulting maximum gap was measured with a calibrated digital microscope. From the distance and the beam dimensions (length: 150 mm, thickness: 0.3 mm), the stress was determined using Stoney’s equation [23] with a measurement uncertainty of 0.06 GPa.

#### 2.1.5. Thermal Decomposition of the SM

The thermal decomposition of QPAC 40 was carried out in argon to prevent oxide formation on the diaphragm and hence, an additional source of stress generation. During the decomposition in an inert gas atmosphere, QPAC 40 went over into a cyclic carbonate vapor leaving minimal ash residues (<10 ppm) [24]. The decomposition process was completed at 350 °C. A stainless-steel pressure container with a cylindrical processing chamber (∅50 mm × 80 mm) was used for the thermal treatment process. The chamber temperature was monitored using an integrated PT1000 temperature probe. A circulating air oven (SNOL 58/350 LSN11, Boldt Wärmetechnik, Biebertal, Germany) was used to heat the container to the desired temperature. Before heating, the processing chamber was purged with argon with repeated evacuation steps (10×) in between (60RVD, Sirio Dental SRL, Meldola FC, Italy). The substrate was heated up to 350 °C with a maximum heating rate of 2.8 °C/min followed by a holding phase of 2 h before the oven was switched off and the substrate was cooled down to ambient temperature with a maximum cooling rate of 1 °C/min. Before the cooling phase started, the purge cycle was repeated to remove all decomposition products from the processing chamber. Before the purge gas entered the chamber, it was heated up to the substrate temperature using a coil heat exchanger in the gas supply line that was situated together with the pressure container inside of the oven.

### 2.2. Diaphragm Inspection and Testing

#### 2.2.1. Diaphragm Surface Shape Characterization

**Figure 5:**
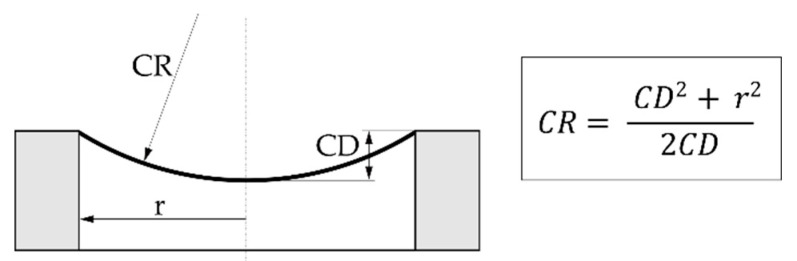
Relationship between the curvature depth (CD) and the curvature radius (CR) of a clamped circular diaphragm with a radius r.

The surface geometry of the diaphragm was characterized by optical inspection and by measuring its curvature depth (CD) before and after the diaphragm was released from the SM. Both procedures were conducted using a Leica DMR light microscope (Leica, Heerbrugg, Switzerland) equipped with different polarization filters. The CD is defined as the vertical distance between the rim and the center of the diaphragm (cf. Figure 5). A frequently used measure for surface curvature is the radius of curvature (CR), which can be calculated from the CD using the formula in Figure 5, where r depicts the radius of the diaphragm.

The CD of the diaphragm was measured by the focus variation (FV) technique using the light microscope. The FV technique uses the small depth of field (DOF) of high-magnification microscope optical systems to obtain the depth information of the object’s surface topography. The DOF for an objective with a magnification of 20 and a numerical aperture of 0.5 is approximately 2 µm [25]. During CD measurement, the diaphragm’s edge and the center were successively brought into focus by the visual observation of the corresponding roughness topography and by moving the vertical micro-positioning stage of the microscope. The corresponding vertical position was read with an optical incremental encoder (AEDB-9140, Broadcom, San José, CA, USA) and a LabView interface (Version 2017, National Instruments, Austin, TX, USA). Despite the very smooth diaphragm surface, an uncertainty of CD measurement below 5 µm was achieved using the FV technique.

#### 2.2.2. Diaphragm Stiffness Measurement

The diaphragm stiffness $K_{m}$ was determined by measuring the diaphragm response to acoustic stimulation using a single-point laser Doppler velocimeter (LDV, CLV-2534, Polytec GmbH, Waldbronn, Germany). The stiffness represents the ratio between the force acting on the diaphragm and the corresponding diaphragm dynamic center displacement $w_{c}$ obtained from the LDV measurement (Equation (1)). The force is the product of the stimulation sound pressure p and the diaphragm’s surface area A.

(1) $$K_{m}=\frac{pA}{w_{c}}$$

**Figure 6:**
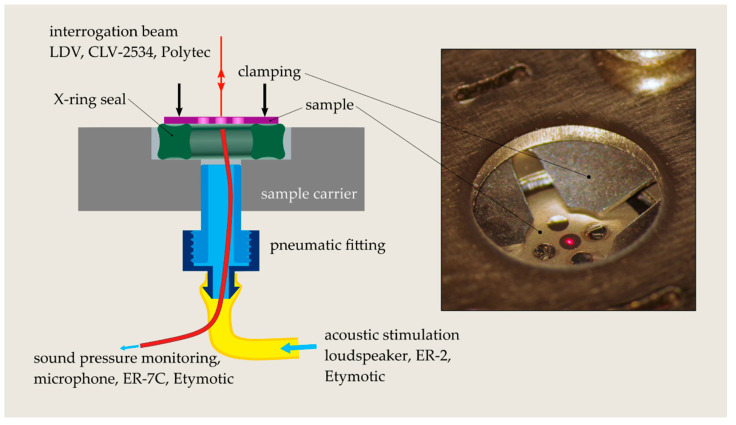
Schematic drawing of the setup for stiffness measurement on the fabricated diaphragms. The method relies on the measurement of the diaphragm response to acoustic stimulation using a single-point LDV system. The microscope image shows a sample with diaphragms clamped down on a rubber seal ring. The interrogation laser beam is positioned in the center of the diaphragm. Three of the five diaphragms are broken and thus sealed with epoxy adhesive.

The interrogation laser beam was directed normal to the front surface of the diaphragm and manually adjusted in the center under a surgical microscope. The acoustic sound impinged the diaphragm from the rear side of the sample (cf. Figure 6). To interface the individual samples with the sound source (ER-2 loudspeaker, Etymotic Research Inc., Elk Grove Village, IL, USA), the substrate was installed on a sample carrier with a pressure port underneath each sample. An airtight coupling between the sample and the carrier was achieved using a rubber X-ring seal underneath each sample and the same clamping plate as employed for sample filling. To minimize deformations of the sample from clamping and hence clamping-induced variations of the diaphragm stress, only a small clamping force was applied on the sample. A Luer-Lock pneumatic fitting was used to interface the sound delivery hose of the loudspeaker system with the pressure port of the sample under investigation. The sound pressure driving the diaphragm was monitored using a microphone (ER-7C, Etymotic Research Inc., Elk Grove Village, IL, USA) optimized for measurements in a human ear canal and thus well suited for measurements inside the closed cavity underneath the sample. The tiny probe tube of the microphone was fed through the pneumatic connector, and the tube’s free end was positioned directly in front of the sample’s rear side (cf. Figure 6). A multi-channel audio analyzer (APx585, Audio Precision Inc., Beaverton, OR, USA) was used to acquire the microphone and the LDV output signals and for outputting the signal that drove the loudspeaker via a power amplifier (RMX 850, QSC Audio Products LLC, Costa Mesa, CA, USA). The data acquisition and signal generation were controlled from a custom-built LabVIEW interface (Version 2017, National Instruments, Austin, TX, USA). To increase the robustness of the stiffness measurement, five frequency points between 1 and 5 kHz were considered to finally obtain an average stiffness value. The LDV signal representing a velocity was filtered using a digital narrow bandpass filter (third-order Butterworth, one-third octave band) before the RMS value was calculated and the velocity divided by the frequency to obtain the vibration amplitude. An excitation sound pressure between 115 and 125 dB SPL was applied to drive the diaphragms in the considered frequency range. With a proper installation of the sample (low clamping force) the setup allowed diaphragm stiffness measurement with an uncertainty smaller than 5% of the measured stiffness value.

#### 2.2.3. Intrinsic Stress Estimation

The intrinsic stress in the diaphragm with a thickness t and radius r was estimated from the measured diaphragm stiffness and using a semi-empirical formula that represents the relationship between the pressure load p acting on a flat, circular diaphragm with intrinsic stress σ and the resulting diaphragm center deflection $w_{c}$ (Equation (2) [26]).

(2) $$\frac{pr^{4}}{Et^{4}}=\left(\frac{16}{3\left(1-\nu^{2}\right)}+\frac{4\sigma r^{2}}{Et^{2}}\right)\left(\frac{w_{c}}{t}\right)+\frac{2.83}{\left(1-\nu^{2}\right)}\left(\frac{w_{c}^{3}}{t^{3}}\right)$$

The Young’s modulus and Poisson ratio of the diaphragm are denoted with E and ν, respectively. The second term on the right side of Equation (2) accounts for a large deflection behavior ($w_{c}\gg t$), which does not prevail for the present application. Hence, the term is neglected. To obtain a formula (Equation (5)) that directly relates the stiffness and the intrinsic stress, the equation was rewritten using the following definitions (Equations (3) and (4)):

(3) $$E^{\prime }=\frac{E}{1-\nu^{2}}$$

(4) $$D=\frac{E^{\prime }t^{3}}{12}=E^{\prime }I$$

Equation (4) describes the flexural rigidity D of a plate, where $E^{\prime }$ denotes the effective Young’s modulus (Equation (3)) and I the area moment of inertia per unit length.

(5) $$\sigma =\frac{1}{4\pi t}\left(K_{m}-\frac{64\pi D}{r^{2}}\right)$$

Most of the fabricated diaphragms represent multi-layer coatings built from Ti ($E_{Ti}=102\;\mathrm{GPa},\;\nu_{Ti}=0.33$) and Pt ($E_{Pt}=172\;\mathrm{GPa},\;\nu_{Pt}=0.38$) with different layer designs and hence a different flexural rigidity $\bar{E^{\prime }I}$. Based on the approach described by Guo et al. [27], the parameter D was calculated for all layer designs considered in the present study (cf. Table 1). The chosen material properties are typical values for bulk Ti (grade 2) and Pt which might deviate from the corresponding properties of sputtered Ti and Pt films. Therefore, the present approach must be regarded as an estimate of the intrinsic stress in a clamped, circular diaphragm with flat surface geometry.

.

## 3. Results

**Figure 7:**
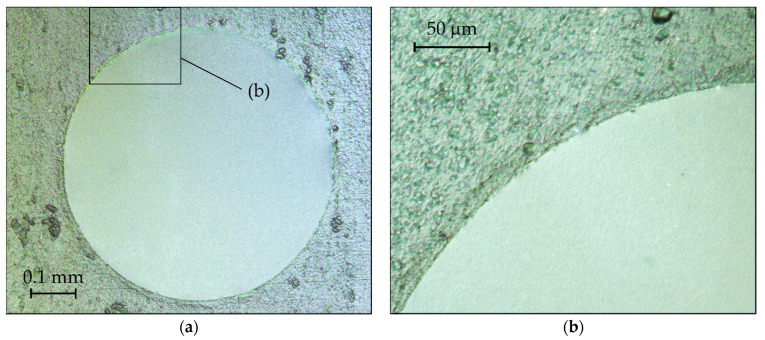
Microscope images of a typical Ti/Pt diaphragm supported by a commercial pure cold-rolled Ti plate: (**a**) image taken at a magnification of 100×; (**b**) zoomed view on the edge region of the diaphragm (magnification 200×).

Figure 7a,b shows microscope images of a typical 1 µm thick and 0.6 mm large Ti/Pt diaphragm fabricated on a 0.3 mm thick Ti substrate using the new developed fabrication technology. The right image is taken at higher magnification and zooms to the edge region of the diaphragm. The diaphragm has a flat geometry and an estimated surface roughness in the nanometer range (specular appearance under visible light). In contrast, the estimated surface roughness of the substrate is in the order of the diaphragm thickness. Preliminary tests have shown that the diaphragms can withstand static pressure loads of up to 1 atm and fulfill requirements for hermeticity according to typical helium leak tests (He leak rate ≤ 1 × 10^−10^ mbar L/s).

**Figure 8:**
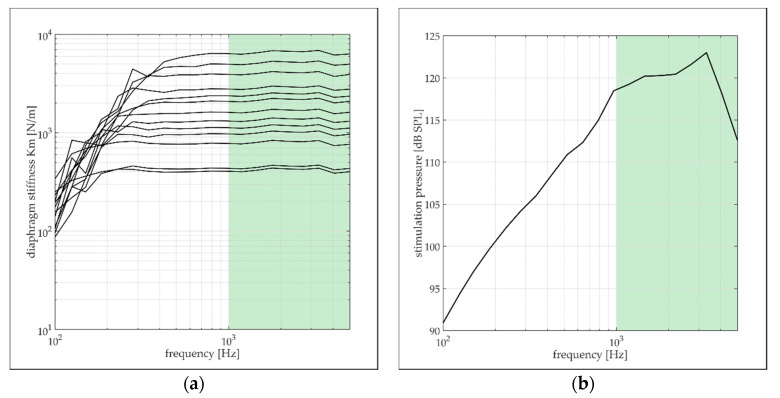
(**a**) Diaphragm stiffness as a function of the stimulation frequency between 100 Hz and 5 kHz. The stiffness data were determined from vibration amplitude measurements (LDV) on the diaphragms driven with acoustic sound; (**b**) sound pressure levels driving the diaphragms between 100 Hz and 5 kHz. The green area in both figures indicates the frequency range considered for averaging the stiffness data over the frequency.

The first eigenfrequency of a clamped circular Ti diaphragm with a 0.6 mm diameter and 1 µm thickness undergoing free vibration in air is located above 30 kHz [28]. Well below the resonance frequency, the diaphragm is purely stiffness driven; i.e., the vibration amplitude is directly related to the mechanical stiffness of the diaphragm. The cavity underneath the sample is sufficiently large to neglect any contribution to the diaphragm’s stiffness. Figure 8a confirms the stiffness-controlled vibration behavior of the diaphragms by the flat frequency response to acoustic stimulation between 800 Hz and 5 kHz (maximum considered frequency). The data represent the diaphragm stiffness calculated according to Equation (1) and determined from LDV measurements on various diaphragms with different stiffness values, which were driven with sound pressure levels as depicted in Figure 8b. At low frequencies, the stiffness starts to deviate from a flat behavior. The falling stiffness is associated with a low signal-to-noise ratio that results from a decaying vibration velocity with decreasing frequency and stimulation strength (cf. Figure 8b). At frequencies below 1 kHz and low vibration velocities, the noise of the LDV increases with decreasing frequency, leading to the measurement of a higher diaphragm response and correspondingly lower stiffness values than expected [29]. Diaphragms with low stiffness show a flat response already above 200 Hz, whereas for high stiffness values, flat behavior arises first above 800 Hz. To ensure robust stiffness measurement even for very stiff diaphragms, only data between 1 kHz and 5 kHz were considered for averaging over the frequency band (cf. green area in Figure 8a,b). The upper frequency bound was set to maintain sound pressure levels above 110 dB SPL and to prevent resonance phenomena in the acoustic supply line of the loudspeaker.

**Figure 9:**
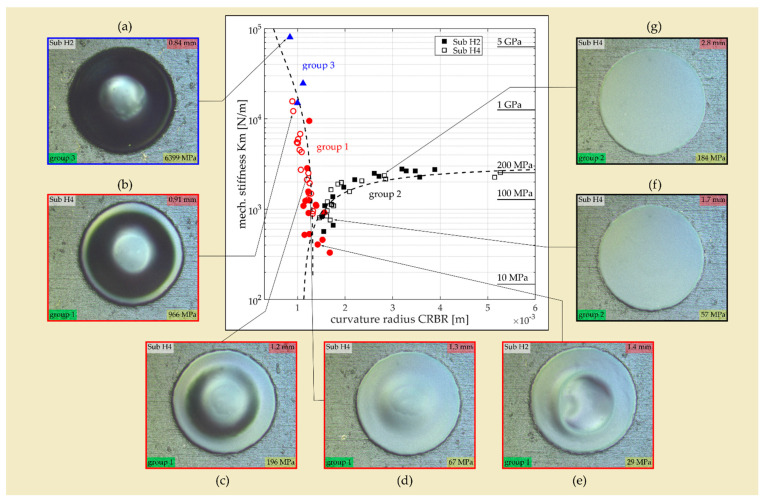
Diaphragm stiffness as a function of the diaphragm’s curvature radius before it is released from the SM (CRBR) for diaphragms of the substrate H2 (filled markers) and H4 (empty markers). Group 1 diaphragms are indicated with red circles, group 2 with black squares, and group 3 with blue triangles. The stiffness-related intrinsic stress is indicated on the right *y*-axis (only valid for group 2 diaphragms). The dashed lines represent trendlines assuming linear behavior if the stiffness is considered in linear space. (**a**–**g**) Optical microscope images showing the diaphragm’s surface geometry (after release) depending on the CRBR. The CRBR and the intrinsic stress values are indicated in the images with a red and yellow background color, respectively. The group name is highlighted green.

The diaphragm fabrication trials revealed that, depending on the surface shape of the SM that defines the shape of the deposited coating, diaphragms with a flat or curved geometry are formed. Moreover, it was observed that the noted surface geometry of the SM has a significant influence on the diaphragm’s intrinsic stress. This dependence is illustrated in Figure 9 by data from diaphragms of two (H2 and H4) of the total seven substrates considered in the present study (cf. Table 1). The coating design of both substrates is identical. It represents a three-layer design composed of two Ti outer layers (425 nm) and one Pt middle layer (150 nm, cf. Table 1) with an overall thickness of 1 µm. The deposition process differs only in the sputter power for Ti, which was increased from 1 kW to 1.5 kW for substrate H4. The figure shows the averaged (over frequency) stiffness data K_m_ of the diaphragms plotted as a function of the curvature radius CRBR that characterizes the shape of the diaphragm before it is released from the SM. The curvature radius was determined from the measured CD data according to the equation depicted in Figure 5. The intrinsic stress calculated from the corresponding stiffness data using Equation (5) is shown on the right *y*-axis of Figure 9. The stress data are only valid for diaphragms with a flat surface geometry.

Diaphragms with a concave shape with high curvature before they are released from the SM (i.e., small CRBR) show very high stiffness. The stiffness rapidly drops with increasing CRBR and reaches minimum values for CRBRs of approximately 1.3 mm. With further increases in the CRBR value, the stiffness starts to rise again but with a smaller slope than for CRBRs below 1.3 mm. Above a CRBR of 3 mm, the stiffness takes a constant value of approximately 2500 N/m.

Considering the diaphragm’s surface geometry after it is released from the SM allows the diaphragms to be classified into three groups. Group 2 represents the diaphragms that are flat after the release process (cf. Figure 9f,g). These diaphragms arise when the CRBR is higher than 1.3 mm. In group 3, the diaphragms remain curved with a concave shape even after they are released from the SM (cf. Figure 9a). They exhibit very high stiffness, which mainly originates from the reinforcement effect associated with a spherical thin-wall geometry. Finally, group 1 contains all diaphragms with a surface geometry that describes an intermediate state between a flat and a curved shape (cf. Figure 9b–e). Within that group, the diaphragm stiffness decreases with smaller CD (after release) and as the fraction of the flat diaphragm surface increases. The lowest stiffness values are reached at the transition between group 1 and group 2. It has to be emphasized that the estimated stress values are only valid for flat diaphragms (group 2). Considering the group 2 diaphragms (flat surface geometry), the varying stiffness with CRBR can be only attributed to varying intrinsic stress. The data of group 2 diaphragms indicate that the intrinsic stress in the diaphragm can be significantly reduced (from approximately 200 MPa to 30 MPa) if the surface geometry of the SM is adjusted for an optimum CRBR (≈1.3 mm) during the filling process.

**Figure 10:**
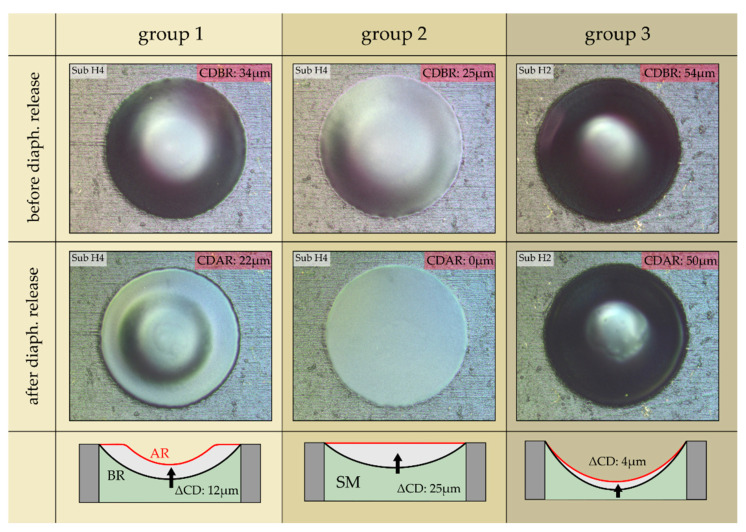
Optical microscope images of typical diaphragms of the three groups before (first row) and after (second row) they are released from the SM. The corresponding curvature depth is indicated with a red tag in the images. The bottom row shows schematic illustrations of the diaphragm profile geometry of each group before (BR) and after release (AR). Dimensions do not scale.

From Figure 9, it is obvious that the diaphragms of group 2 change the surface geometry while they are released from the SM. As depicted in Figure 10, this is also true for the diaphragms of the two other groups. The figure shows optical microscope images of representative diaphragms of each group before (first row) and after (second row) the release process. The corresponding CD is indicated by the red tag in each image. Before release, all diaphragms show a curved geometry with a CDBR between 25 µm and 54 µm, whereas after release the diaphragms adopt a geometry with smaller curvature. The curvature change (ΔCD) provoked by the release process varies in a broad range with the largest change for the group 2 diaphragm (from 25 µm to 0 µm) followed by group 1 (from 34 µm to 22 µm). For the diaphragm of group 3, only a small change in the CD was identified (from 54 µm to 50 µm).

**Figure 11:**
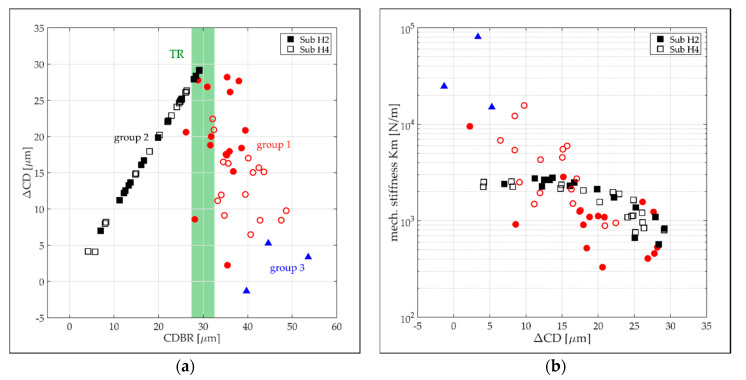
(**a**) Release induced change of the diaphragms’ CD (ΔCD) as a function of the corresponding CD value before the release process (CDBR). Considered are again data of the diaphragms on substrate H2 (filled markers) and H4 (empty markers). The data are color-coded depending on the diaphragm group. The green shaded area represents the transition region between group 1 and 2 diaphragms; (**b**) mechanical diaphragm stiffness plotted versus ΔCD.

In Figure 11a, the ΔCD data of all diaphragms on substrate H2 and H4 are plotted against the CD before they are released from the SM. The data are again color-coded according to the three diaphragm groups. The effect of the ΔCD on the diaphragm stiffness is illustrated in Figure 11b. The ΔCD of group 2 diaphragms increases linearly with increasing CDBR up to the transition region (TR) where the group 2 diaphragms go into group 1. The TR is associated with the occurrence of the lowest intrinsic stress or stiffness, respectively. From there, the ΔCD starts to drop rapidly with further increases in the CDBR up to the diaphragms of group 3 where only a small ΔCD was identified. The high spreading of the group 1 data is explained with the uncertainty of CD measurement of 5 μm and the less defined surface geometry (curved and flat parts) after diaphragm release compared with a flat (group 2) or fully curved (group 3) diaphragm geometry. We assume that the intrinsic stress in the diaphragm formed during the deposition process is the driving force for the ΔCD causing full or partial stress relief while the diaphragm is released from the SM. Despite the expected full stress relief for the diaphragms of groups 1 and 3, they exhibit high stiffness as a result of the non-flat surface geometry. With an increasing CDBR and hence higher diaphragm stiffness, the stress relief mechanism loses its effectiveness as a driving force for ΔCD.

**Figure 12:**
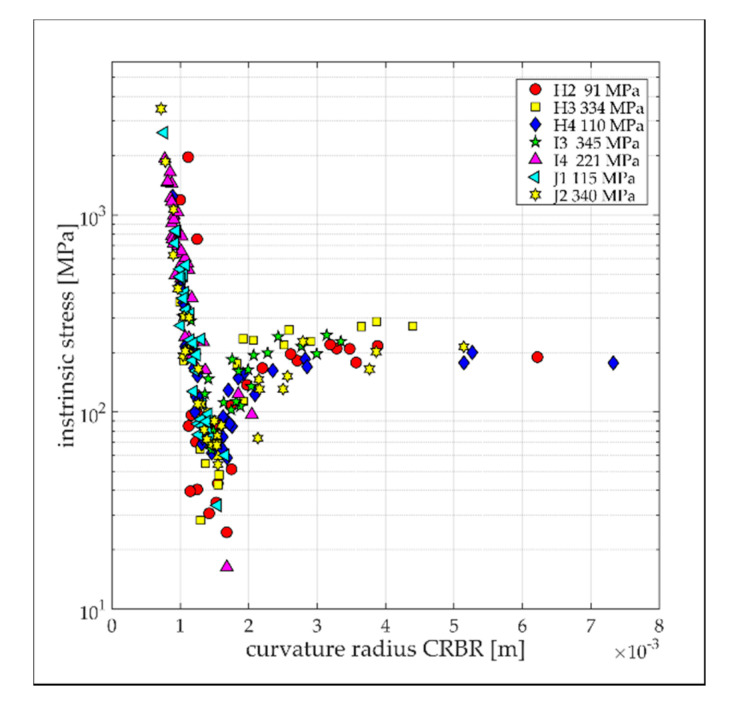
Intrinsic stress as a function of the CRBR for different mono- and multi-layer coatings. The stress values are only valid for diaphragms with a flat surface geometry (group 2, CRBR > 1.3 mm). The stress levels of the coatings deposited on SCS beams are indicated in the legend.

Different Ti/Pt multi-layer coatings were tested to reduce the intrinsic stress in the diaphragm by optimizing the coating design and the sputtering process. The goal was to verify if, in combination with the stress relief mechanism discussed above, nearly stress-free diaphragms can be fabricated. The coatings that were tested so far showed a tensile stress on SCS beams (reference method for stress characterization) between 90 and 345 MPa (cf. Table 1). The corresponding stress values obtained from measurements of the diaphragm stiffness are shown in Figure 12. The data are again plotted as a function of the CRBR to illustrate the effect of the different coating designs on the stiffness of diaphragms of the different groups. The stiffness of group 1 and 3 diaphragms (CRBR < 1.3 mm) is not affected by the coating design at all. This outcome appears to be reasonable, as the stiffness of these diaphragms is mainly determined by the curved geometry. Because of the stress relief mechanism, the contribution of intrinsic stress on the diaphragm’s stiffness should be negligible. In contrast, the flat diaphragms (group 2) show clear variations in intrinsic stress between the different substrates. To compare the diaphragm stress with the measured stress on the SCS beams, diaphragms with CRBRs larger than 3 mm (stress relief mechanism should have a small effect) were considered. On substrate H3, similar stress levels were observed as on the corresponding SCS stripe, whereas the diaphragms of the substrates I3 and J2 showed considerably lower stress than the corresponding reference values (>340 MPa). In contrast, the low reference stress in the range of 100 MPa was not found on the diaphragms. The deviations are not surprising. In contrast to the SCS beams, the diaphragms were deposited on a polymer SM which could influence the crystal growth during deposition. It is also conceivable that, compared with SCS, a different adhesion between the coating and SM influences the evolution of intrinsic stress during the deposition process that differs from the adhesion on SCS and causes stress relief. At last, the diaphragms form a free-standing, clamped plate structure with boundary conditions that could influence stress formation. Considering the group 2 diaphragms closer to the TR, stress variations between the substrates could not be assigned to the specific sputter process but rather to the strong dependence of the CRBR on the diaphragm stress within the TR.

## 4. Discussion

The present study introduces a new fabrication process for micron-thick Ti and Ti/Pt multilayered diaphragms. In contrast to typical MEMS fabrication technology for diaphragm fabrication, the introduced fabrication process does not rely on anisotropic dry etching, which is not well established for the processing of Ti substrates. In addition, dry etching in combination with a sacrificial material layer as an etch stop requires highly polished substrates. Such substrates made from Ti are not off-the-shelf products such as silicon wafers; they have to be custom-made by chemical mechanical polishing (CMP), a process that is also not very well established for Ti in typical MEMS fabrication facilities.

We could show that the introduced fabrication process seems to be compatible with unpolished substrates with a surface roughness similar to the thickness of the diaphragm. The possibility of using untreated Ti substrates lowers the fabrication complexity and the associated costs but also provides more freedom during the design process of the diaphragm support structure (i.e., 3D geometries such as tubes, etc.). We assume that the higher roughness of the substrate might even improve the strength of the interface between the diaphragm and the support, providing a kind of interdigitation mechanism with an increased contact area at the interface. Currently, a study is ongoing to better understand and assess the interface between the diaphragm and the support using more dedicated inspection techniques, such as scanning and transmission electron microscopy in combination with static pressure loading and hermeticity tests.

Another advantage of the new diaphragm fabrication technology is the ease of defining a nonplanar surface shape of the diaphragm before it is released from the SM. Currently, the SM is applied as a solution with low viscosity where surface tension forces enforce the formation of a spherical surface shape with a curvature radius defined by the filling state. During the thermal decomposition of the SM, the diaphragm tends to adopt a flat surface geometry. It is assumed that the mechanism is driven by tensile intrinsic stress in the coating and results in full or partial stress relief while the diaphragm is released from the SM. A similar stress relief mechanism was reported by Wang et al. [30] on single deeply corrugated diaphragms (SDCD) fabricated from polysilicon on a silicon substrate. The design contained a flat membrane with suspending sidewalls forming a deep corrugation. They observed a zero-pressure deflection (cf. ΔCD) of the thin-wall structure when it was released from the sacrificial layer. Compared with a flat diaphragm design with equal intrinsic stress, the SDCD showed the considerably higher mechanical sensitivity of the transducer, which was increasing with higher corrugation depths. Wang et al. concluded that the suspending side walls have a relieving effect on the intrinsic stress in the sensing membrane. The group 2 diaphragms of the present study can be regarded as an SDCD before they are released from the SM. In contrast to the design of Wang et al., the group 2 diaphragms undergo a larger zero-pressure offset during their transition into a flat geometry state. Similar zero-pressure offset values as reported by Wang et al. were only observed on diaphragms of group 3 with CDBR values (cf. corrugation height) larger than 40 µm. Such diaphragms show very high stiffness as a result of the curved geometry, whereas the SDCD design benefits from the flat and stress-free section that is used for pressure sensing.

**Figure 13:**
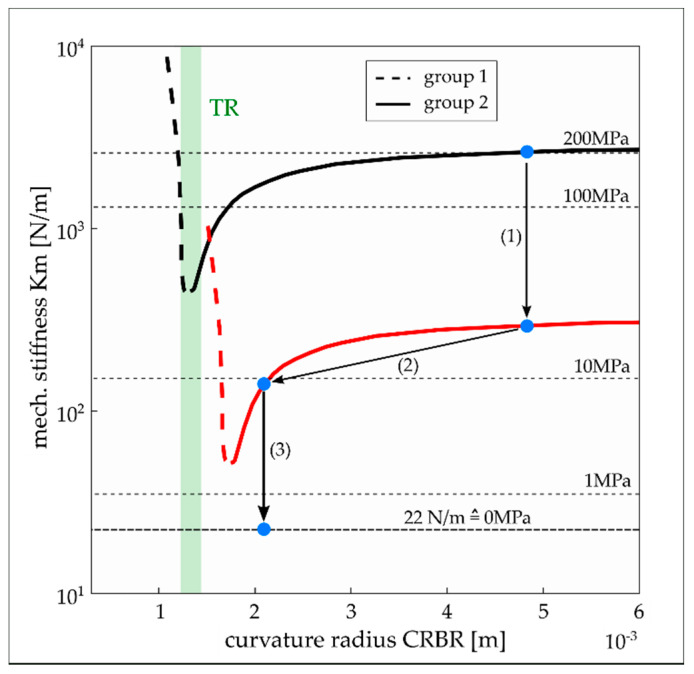
A possible strategy for the fabrication of stress-free diaphragms illustrated in a Km-CRBR plot: (1) Ti/Pt multi-layer coating optimized for low stress and high mechanical strength; (2) stress relief mechanism as a result of the adjustment of an optimum diaphragm surface shape before it is released from the SM; (3) thermal annealing. Remark: The stress values are valid for a diaphragm with 0.6 mm diameter and 1 mm thickness and made from an H2 coating. For zero intrinsic stress, the diaphragm exhibits a stiffness of 22 N/m.

The CRBR transition region (TR) between group 1 and group 2 diaphragms (CRBR ≈ 1.3 mm) is narrow and is characterized by the occurrence of lowest stress values that increase rapidly on both sides of the TR (cf. Figure 13). The limited accuracy of CD measurement using the focusing technique and the number of available data do not currently allow a more precise characterization of the TR, such as the width and potential shifts in CR depending on the stress level of the coating. The coatings considered in the present study were mainly designed to obtain low stress. Hence, the largest stress differences between the coatings are not larger than 100 MPa (considering the stress of group 2 diaphragms at high CRBRs). It is expected that if the stress in the coating is further reduced, the TR will shift to higher CRBRs (cf. red curve in Figure 13). Higher stress levels should shift the TR in the opposite CRBR direction. Although diaphragms within the TR could benefit from a high stress relief, the steep gradients on both sides would make the control of the fabrication process difficult. The design point on the Km-CRBR characteristic curve must be situated on the right side of the TR (group 2 diaphragms) in a region where the slope of the curve complies with the allowable part-to-part variation. To shift the design point as close as possible to the TR and hence benefit from a high stress relief, the process for applying the SM on the substrate (i.e., cavities) needs to be optimized concerning a precise adjustment of the desired CRBR or CDBR, respectively.

However, to obtain diaphragms nearly free of intrinsic stress, the stress relief mechanism must be combined with other technologies for stress reduction in the coating, such as sputter process parameter optimization, multi-layer coatings, and thermal annealing. A potential multi-step strategy is depicted in Figure 13. In the first step, the intrinsic stress in the coating must be reduced by an optimization of the sputtering process and by the use of a multi-layer coating design. The minimum stress that can be achieved will result from a trade-off behavior between the stress and the mechanical strength of the diaphragm (pores, voids, and defects). Using the stress relief mechanism presented in the present study would be the second step to further reduce the stress in the diaphragm. Here, the potential of stress reduction depends on the aforementioned fabrication process considerations and the characteristics of the Km-CRBR curve at low-stress level values, which are currently only estimated. Thermal annealing is considered as the third step to end up with a stress-free diaphragm. Annealing could be an efficient way to reduce stress in a PD because the protective enclosure of the transducer in which the PD is integrated is free of electronic parts that would limit the process to low annealing temperatures. Currently, a study is ongoing to evaluate a Ti/Pt multi-layer coating with low stress, high mechanical strength, and long-term hermeticity. In addition, thermal annealing shall be applied on the diaphragm samples to verify if it is suitable to fully eliminate the remaining residual stress.

As reported in Prochazka et al. [6], the diaphragm fabrication technology was already applied on the sound receptor of the ICAR, which represents a tube-like structure with a rectangular cross-section and multiple PDs arranged within the tip region on two opposite sides (cf. Figure 1b). The feasibility of critical process steps such as the more complex filling with SM and the release of gas products through a micro-channel during the thermal decomposition of the SM were verified by several tests. Further process optimizations are ongoing. In addition to the aforementioned evaluation of a process for PDs free of intrinsic stress, the filling process needs further optimization to make the process faster and more repeatable. Currently, a polymer solution with only 20 weight percent of QPAC is used as an SM for filling the cavities. The process must be conducted in multiple steps because most of the added QPAC volume is lost during the curing of the solution. Using a polymer melt instead of a solution might considerably reduce the required filling cycles, but is also associated with a more complex dispensing system required for a melt instead of a solution. Further improvement of the filling efficiency but also precision and repeatability might be achieved by an automated filling process using an appropriate robot system.

## 5. Conclusions

Overall, our results clearly show that using thin film deposition techniques on a low-temperature decomposable polymer as a SM seems to be a promising technology for the fabrication of thin-walled metallic encapsulations in packaging solutions. In particular, the technology is beneficial if the packaging solution incorporates substrate materials and a complex 3D geometry that are not in line with the typical requirements of classical MEMS fabrication processes such as the use of polished silicon 2D substrates. The present study demonstrates the capability of the technology for the fabrication of 1 µm thick and sub-millimeter large Ti (or Ti/Pt multi-layer) diaphragms on an unpolished commercially pure Ti substrate. Currently, the SM is applied on the substrate using adhesive dispensing technology, which suits application with a non-planar surface shape of the deposited diaphragm before it is released from the SM well. After the removal of the SM, the diaphragm takes a surface shape with a smaller curvature, causing a certain relief of intrinsic stress. We could determine a curvature range with the largest stress relief from stiffness measurements on numerous diaphragms with different surface curvatures before their release.

A next step would be to further optimize the fabrication process to obtain robust and hermetic diaphragms with near-zero intrinsic stress. These PDs are required for the encapsulation of implantable acoustic transducers, such as for a fully implantable cochlear implant system. Further research and development work is required to explore further advantages but also limitations of the present technology in comparison with the classical MEMS technologies for diaphragm fabrication based on bulk and surface micromachining. The focus will be laid on the mechanisms controlling the interface between the PD and the unpolished substrate surface and on alternative processes for the dispensing of the polymer SM to make the process more efficient, precise, and repeatable.

## Figures and Tables

**Table 1:** Coating designs, sputter process parameters, and material properties of the diaphragms considered for the present study. For all sputter runs, argon at 55 sccm (0.9 mTorr) was used as a sputtering gas.

Sample No.	Coating	Number of Layers [#]	Power Ti/Pt Target [W]	Intrinsic Stress on SCS [MPa]	Flexural Rigidity [Nm × 10^−9^] ^1^
H2	425 nm Ti/150 nm Pt/425 nm Ti	3	1000/200	91	9.49
H3	460 nm Ti/90 nm Pt/460 nm Ti	3	1000/200	334	9.76
H4	425 nm Ti/150 nm Pt/425 nm Ti	3	1500/200	110	9.49
I3	227 nm Ti [30 nm Pt/227 nm Ti] 3	7	1000/200	345	9.76
I4	82 nm Ti [10 nm Pt/82 nm Ti] 10	21	1000/200	221	10.14
J1	300 nm Ti [10 nm Pt/60 nm Ti] 10	21	1000/200	115	9.94
J2	1000 nm Ti	1	1500	340	9.47

^1^ The flexural rigidity was calculated based on the approach described by Guo et al. and assuming the following material properties for Ti and Pt: $E_{Ti}=102\;\mathrm{GPa},\;\nu_{Ti}=0.33,\;E_{Pt}=172\;\mathrm{GPa},\;\nu_{Pt}=0.38$
